# New scheme for braiding Majorana fermions

**DOI:** 10.1088/1468-6996/15/6/064402

**Published:** 2014-11-25

**Authors:** Long-Hua Wu, Qi-Feng Liang, Xiao Hu

**Affiliations:** 1 International Center for Materials Nanoarchitectonics (WPI-MANA), National Institute for Materials Science, Tsukuba 305-0044, Japan; 2 Graduate School of Pure and Applied Sciences, University of Tsukuba, Tsukuba 305-8571, Japan; 3 Department of Physics, Shaoxing University, Shaoxing 312000, People’s Republic of China

**Keywords:** topological superconductor, Majorana fermion, non-Abelian statistics, Bogoliubov–de Gennes equation, quantum computation

## Abstract

Non-Abelian statistics can be achieved by exchanging two vortices in topological superconductors with each grabbing a Majorana fermion (MF) as zero-energy quasi-particle at the cores. However, in experiments it is difficult to manipulate vortices. In the present work, we propose a way to braid MFs without moving vortices. The only operation required in the present scheme is to turn on and off local gate voltages, which liberates a MF from its original host vortex and transports it along the prepared track. We solve the time-dependent Bogoliubov–de Gennes equation numerically, and confirm that the MFs are protected provided the switching of gate voltages for exchanging MFs are adiabatic, which takes only several nano seconds given reasonable material parameters. By monitoring the time evolution of MF wave-functions, we show that non-Abelian statistics is achieved.

## 1. Introduction

Majorana fermions (MFs) are particles equivalent to their own anti-particles [1]. Great research effort has been devoted to searching for MFs in condensed matter systems in the last decade since the particles can be used for constructing topological qubits and robust quantum computation [2–5]. Systems that can realize MFs include Pfaffian 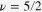 fractional quantum Hall state [6], chiral p-wave superconductors (SCs) [7], one-dimensional (1D) spinless SCs [8] and superfluidity of cold atoms [9, 10]. Recently, two heterostructure systems are suggested as possible MF hosts, one is the 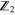 topological insulator in proximity to s-wave SC and ferromagnetic insulator (FMI), the other is the spin–orbit coupled semiconductor (SM) sandwiched by s-wave SC and FMI (SC/SM/FMI) [11–17]. Both systems are effective two-dimensional (2D) 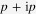 topological SCs, characterized by nonzero Chern numbers (or TKNN numbers) [18, 19]. 1D SM nanowires with spin–orbit coupling in proximity to s-wave SC under magnetic field has also been investigated both theoretically and experimentally for realizing MFs [20–27]. Lately, a promising signal of MFs has been captured in the device of InSb nanowire/s-wave SC [27]. In these heterostructure systems, the rare p-wave pairing are superseded by the interplay between proximity-induced s-wave superconductivity and strong spin–orbit coupling.

It was revealed that MFs appear inside vortex cores in topological p-wave SCs [7], and that non-Abelian statistics can be achieved by exchanging positions of vortices hosting MFs [28]. However, it is difficult to manipulate vortices in experiments, which may hinder the realization of this genius idea. To circumvent this problem, MFs at sample edges of topological SCs have been considered [31]. Making use of their topological properties, edge MFs can be braided with desired non-Abelian statistics by tuning point-like gate voltages on links among topological SC samples. In order to make the edge MFs stable, one needs to embed the device into a good insulator. The size of topological SCs should also be chosen carefully since the wave-functions of edge MFs become too dilute for large samples, which makes edge MFs fragile due to excited states with small energy gap.

**Figure 1.**
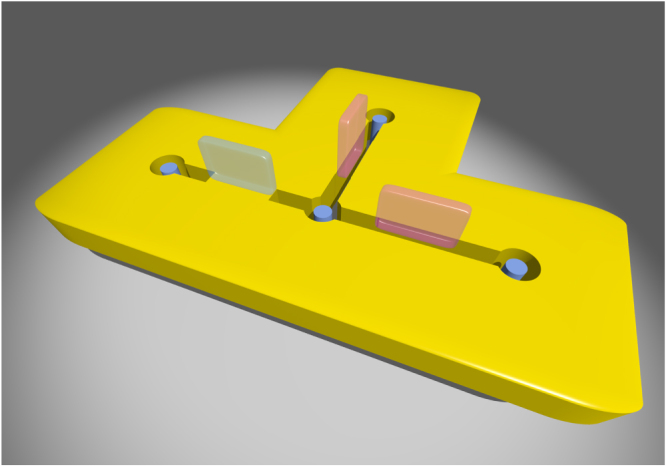
Schematic device setup for braiding MFs at vortex cores. There are four holes in SM layer (yellow platform) with one superconducting vortex (blue cylinder) pinned right beneath each of them. The electrodes at high-voltage states (pink rectangular prisms) prohibit electron hoppings in the regions below them, and thus connect effectively the holes; the blue rectangular prism denotes an electrode at zero-voltage state.

In this work we concentrate on MFs grabbed at vortex cores. We demonstrate that the core MFs can be liberated from vortex cores, transported and braided by applications of local gate voltages. The scheme takes fully advantages of SC/SM/FMI heterostructure in the way shown schematically in figure 1: four holes are punched in the SM layer, and three electrodes are placed above the small regions between holes; gate voltages can be applied via the electrodes, and the ones at high voltage state (pink rectangular prisms in figure 1) connect holes by killing electron hoppings locally; one vortex (blue cylinders in figure 1) is induced and pinned right beneath each hole in the common SC substrate (SC and FMI are not shown explicitly for clarity). The key observation is that the geometric topology of the SM layer can be controlled by local gate voltages, and that when even number of holes are connected, core MFs fuse into quasi-particle states with finite energies, while one core MF exists when odd number of holes are connected. Core MFs can then be liberated from and transported among vortices with a sequence of turning on and off gate voltages at the electrodes. By solving the time-dependent Bogoliubov–de Gennes (TDBdG) equation upon adiabatic tunings of gate voltages, we simulate the time evolution of MF wave-functions and confirm that the braiding of core MFs obeys non-Abelian statistics. Finally, we compare briefly our scheme of manipulating MFs with other proposals in both topological 2D 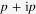 SCs and 1D nanowire networks and show advantages of our scheme.

## 2. Topological superconductivity

### 2.1. Tight-binding model

We start from a tight-binding Hamiltonian on square lattice for a SM with Rashba-type spin–orbit coupling in proximity to a FMI [31]

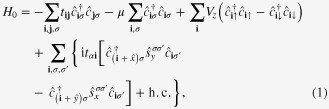
where 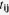 and 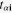 are the nearest-neighbor hopping rates of electrons with preserved and flipped spin directions respectively; *μ* and *V*_*z*_ are chemical potential and strength of Zeeman field respectively; 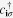 creates one electron with spin *σ* = ↑, ↓ at lattice site 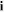; 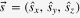 are the Pauli matrices for spin; 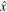 and *ŷ* are unit lattice vectors along *x* and *y* directions.

The proximity-induced superconductivity in SM is described by

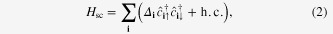
where 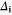 is the pairing potential at site 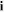. The Bogoliubov–de Gennes (BdG) equation of total Hamiltonian 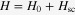 is given by

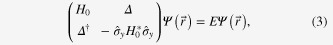
where 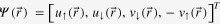 is the Nambu spinor, and 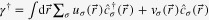 is the quasi-particle operator. For a typical square sample with 200 × 200 sites, the Hamiltonian matrix in (3) has a dimension of 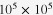. In most cases only several states near *E* = 0 are relevant as far as MFs are concerned, for which a powerful numerical technique is available [32].

### 2.2. Hamiltonian in momentum space and Chern number

**Figure 2.**
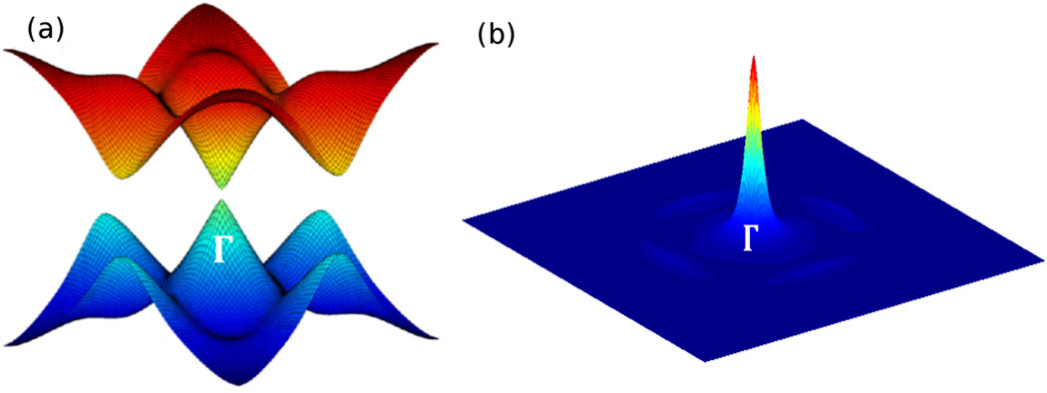
(a) Dispersions of two bands close to Fermi level. (b) Berry curvature 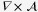 of occupied bands in the first Brillouin zone.

Before solving the BdG equation in a finite sample, we reveal the condition for achieving topological superconducting state in an infinite system. We transform Hamiltonian (1) into momentum space by expanding the annihilation operator as

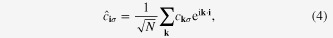
where *N* is the total number of lattice sites, 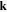 is the crystal momentum. We then obtain the momentum space Hamiltonian on the basis 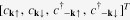


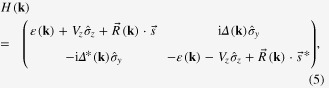
where 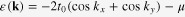 and 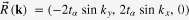. Diagonalizing Hamiltonian (5), we obtain the energy dispersion (see figure 2(a))

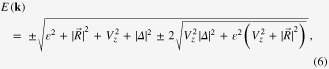
with gap closed at

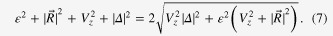
By squaring both sides of (7), we find that the conditions to close the bulk energy gap are

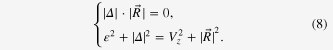
Equation (8) can be fulfilled only when 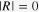 (since 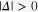). The 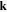-points with 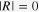 are 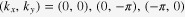 or 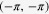 in the first Brillouin zone 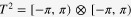. Combining the two conditions in (8), we arrive at the critical *V*_*z*_ to close the bulk energy gap at 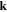-points where 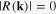


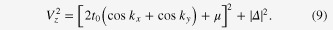



To investigate whether the system is topologically nontrivial when the bulk gap closes and reopens while tuning the Zeeman field *V*_*z*_, we evaluate the Chern number [18, 19]

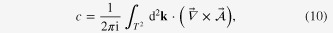
with Berry connection 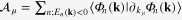 (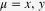), where summation is taken for all occupied bands 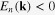, and 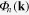 is the wave-function of band *n* at momentum 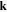. Integrating the Berry curvature 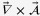 shown in figure 2(b) over the Brillouin zone [33], we find that *c* = 1 for 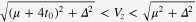 with 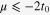. The nonzero Chern number is attributed to the topologically nontrivial energy gap at *Γ* point 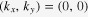 while those at 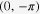, 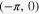 and 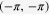 remain trivial [16, 20, 34]. The nonzero Chern number indicates that the system is topological and thus may support MFs. The above result is consistent with that obtained by the 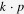 approximation around *Γ* point [34].

## 3. Manipulations of core MFs

### 3.1. Core MFs

**Figure 3.**
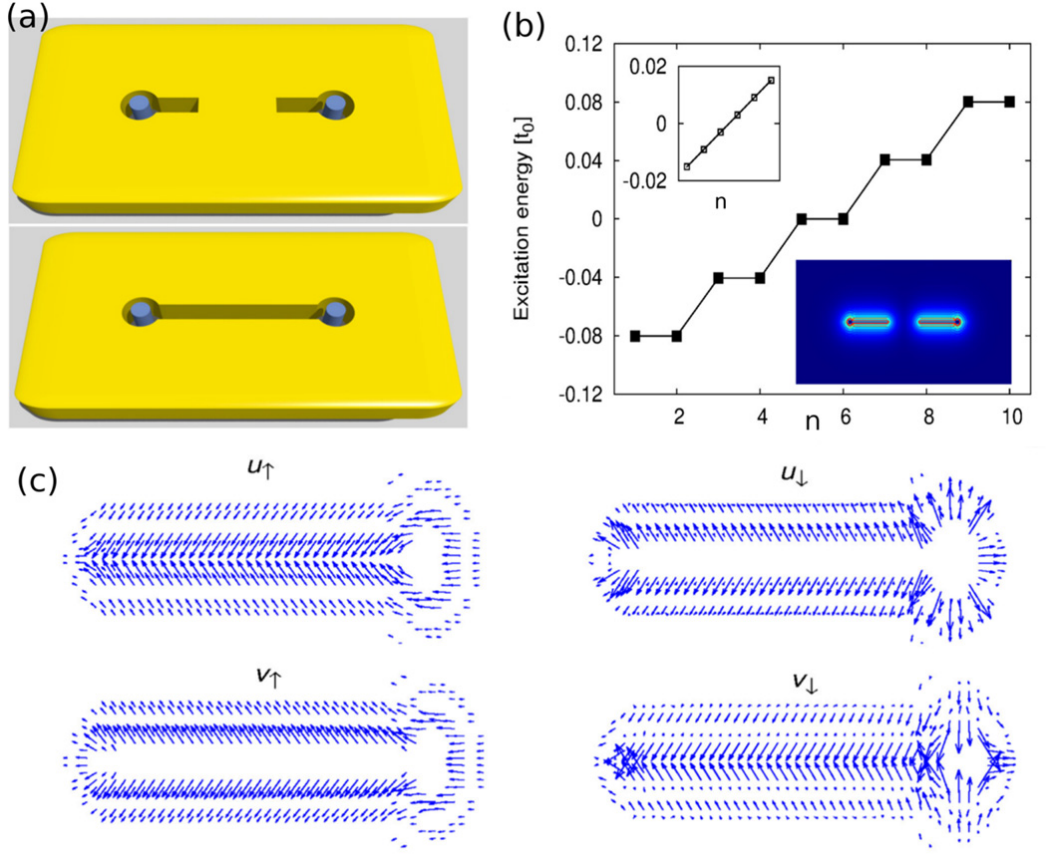
(a) System of two holes and two vortices of positive vorticity with holes isolated (top panel) and connected (bottom panel). (b) Energy spectrum of several low-energy excitations at vortex cores when the holes are isolated with *n* the serial number of eigenstates with energies close to zero. Upper inset: energy spectrum of excitations at the sample edge. Lower inset: distributions of zero-energy quasi-particles. Results are for 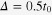, 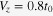, 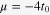 and 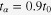 with a sample of 400 × 200 sites. (c) Four spinor components of zero-energy states at the right hole, with the length and azimuth angle of vectors denoting the amplitude and phase of spinor fields respectively.

Now we study a finite sample with two separated holes in the SM and two vortices of positive vorticity pinned right beneath them (see top panel of figure 3(a)). The typical size of a sample is 600 × 300 nm^2^, which is divided into 400 × 200 square grids, corresponding to the Hamiltonian matrix of dimension 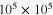 in (3). By solving the BdG equation for this case, we obtain the energy spectra of excitations and eigen-functions. Two zero-energy states are found at the holes, whereas no such state at the edge, as shown in figure 3(b). We examine the four spinor components of the zero-energy states, and find 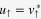 and 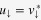 (displayed explicitly in figure 3(c) for the right hole), which results in 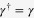, indicating that the two zero-energy states are Majorana states.

It should be noticed that the excitation energy gap at vortices is about four times larger than that at the edge (see inset of figure 3(b)), which makes the core MFs more stable than edge MFs [31]. On the other hand, because the minigap associated with Andreev bound states at superconducting vortex core proximity-induced in SM is roughly 
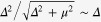
 with a small Fermi energy 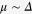 [35], the influence from Andreev bound states to the core MFs can be neglected. It is in contrast to the case of SC exposed to vacuum where 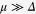 and thus the minigap is small in order of 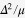.

Next, we impose a point-like gate voltage on the region between the two holes to prohibit direct hopping of electrons by lifting the on-site energy there as in the bottom panel of figure 3(a) (see also figure 1). This merges effectively the two isolated holes into a unified one. Solving the BdG equation for this case, there is no zero-energy quasi-particle, since the combined hole includes two vortices [36].

### 3.2. Transportation of MFs

**Figure 4.**
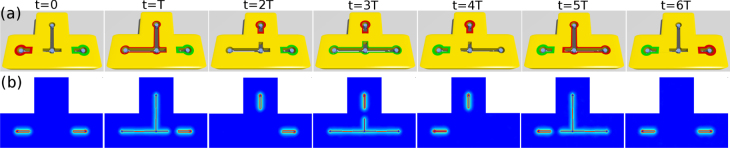
(a) Schematic diagram for braiding the two MFs at the left and right holes, colored by red and green respectively. (b) Distributions of the wave-functions of MFs obtained by solving TDBdG.

Based on the above result that two MFs can fuse into finite energy excitations by connecting two holes, we can design a way of liberating and transporting a MF from one vortex to another. Initially, the top and middle holes are connected together while the leftmost one is isolated and hosts a MF (see figure 4(a) with *t* = 0). We then combine these three holes by applying gate voltages on the region between the left and middle ones, which causes the MF to spread itself over the unified hole including three vortices (see figure 4(a) with *t* = *T*). Finally, the MF is moved totally to the top by disconnecting the top hole from others (see figure 4(a) with 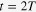). It is noticed that the collapsing of MF wave-function on the top hole is a topological property, and is impossible for electrons and photons. The energy gap remains finite during the whole processes, which guarantees topological protections on the MF state.

### 3.3. Braiding of MFs

Being able to transport one MF from one hole to another, we extend the scheme to interchange positions of two core MFs in the system shown in figures 1 and 4. Following the above transportation procedures, we further move the green MF from the right hole to the left one during 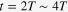 in the same way as above. Finally, the red MF stored temporarily at the top hole is transported to the right one in the period 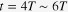. After the sequence of switching processes, the system comes back to the original state with the red and green MFs exchanged.

In order to keep the topological protection, we need to manipulate the gate voltage in an adiabatic way. The reason is that the MF states have certain probabilities to be excited to higher energy states for non-adiabatic processes, which results in the collapsing of the whole braiding scheme. Given reasonable material parameters, the typical time for a single round of braiding is estimated to be within several nano seconds, which is sufficiently short time for practical applications.

### 3.4. TDBdG equation

In order to investigate the impact of position exchanging to MF states, we monitor the time evolution of MF wave-function 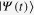 by solving the TDBdG equation numerically

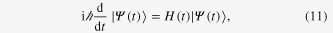
where *H*(*t*) is given in (3) and depends on time in terms of the hopping rates 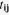 and 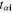 at the regions between holes, which are tuned adiabatically by the local gate voltages [31].

Even with the powerful computation resources available these days, it is still hopeless to tackle this problem by directly diagonalizing the Hamiltonian *H*(*t*) of dimension 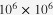 for each time instant. Fortunately, it has been revealed that when the exponential operator is expanded by the Chebyshev polynomial

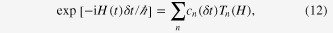
the coefficient 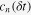 decreases with *n* exponentially fast for small 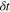 [37]. Therefore, only several leading terms *c*_*n*_ are necessary for a sufficiently accurate estimate of 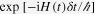. Moreover, the Chebyshev polynomials can be constructed based on the recursive relation 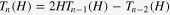, which reduces the computation cost further. In this way, the TD wave-function of the MFs can be obtained efficiently with sufficient accuracy in an iterative fashion 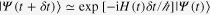.

**Figure 5.**
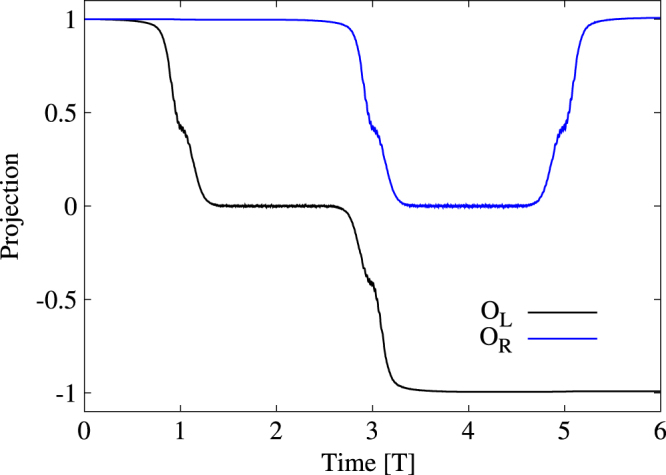
Projections of the MF wave-function 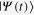 obtained by TDBdG onto the initial states 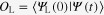 and 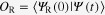.

The wave function at time *t* = 0 is defined as 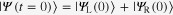 with 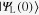 and 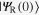 the zero-energy states at left and right holes, respectively, as shown in figures 4(a) and (b). We evaluate the projections of 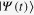 onto the initial states 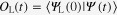 and 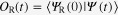 during adiabatical braiding processes and display them in figure 5 for 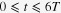. Since four spinor components of MF satisfy 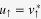 and 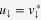 (see figure 3(c)), MF wave function can be simplified to 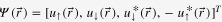. The projections *O*_L_ and *O*_R_ then must be real numbers. The two MFs pick up opposite signs after exchanging their positions, which can be summarized by

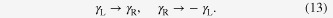
Equation (13) can be written as 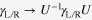 with the unitary matrix 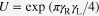. This indicates that the braiding of MFs satisfies non-Abelian statistics [24, 25, 31, 40, 41, 43, 44], as will be shown explicitly below.

## 4. Comparisons with other proposals for braiding MFs

### 4.1. Braiding MFs in 2D p-wave SCs

We begin by reviewing Ivanovʼs model for realizing non-Abelian statistics, which describes low-energy excitations bound to vortices in spinless p-wave SCs [28]. The Hamiltonian is [28–30]

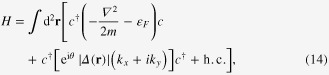
where the first term describes kinetic energy and chemical potential 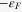, 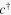 is electron creation operator, and the second term gives pairing function 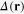 with superconducting phase *θ*, *k*_*x*_ and *k*_*y*_ are electron momenta along *x*- and *y*-axis respectively. The Bogoliubov quasi-particle operator is 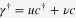, which satisfies the BdG equation

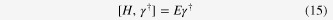
with *E* the eigenenergy of quasi-particle. By taking Hermitian conjugation † at both sides of (15), we have

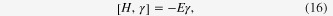
indicating the particle-hole symmetry of BdG equation, i.e. 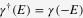. Thus, the zero energy states must be double-degenerate, satisfying self-adjoint condition 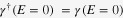, which are MFs.

We first consider how MFs evolve under 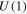 gauge transformation. Since *θ* can be absorbed into fermionic creation and annihilation operators: 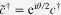 and 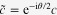, the new Bogoliubov quasi-particle operator is

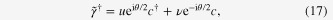
which can diagonalize the gauge-fixed Hamiltonian

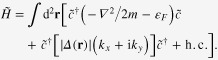
In the case of 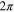 phase changing 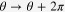, we have 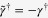, which is an important result that can be employed to achieve non-Abelian statistics.

**Figure 6.**
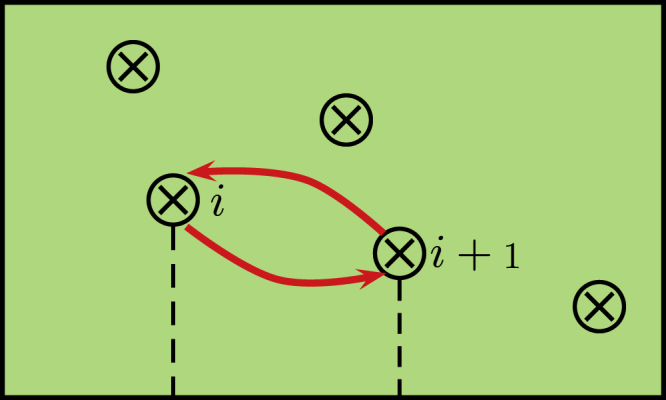
Braiding of vortex *i* and vortex 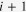 in a 2D spinless p-wave superconductor. Dashed black lines connecting to bottom boundary are cuts for superconducting vortices [28]. Red arrows are braiding loops.

In a 2D spinless 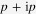 SC with many vortices, superconducting phase *θ* around each vortex can be single-valued apart from a cut (dashed black line in figure 6), where *θ* jumps by 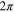. It is then easy to see that the Majorana wave-function bound to a vortex core picks up a sign 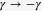 after crossing the cut. By interchanging positions of vortices *i* and 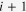 (see red arrows in figure 6), one observes that MF 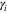 passes through the cut of vortex 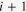 and picks up 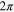 phase (see figure 6), which gives

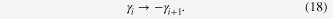
The other Majorana state at vortex 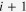 does not cross the cut of vortex *i*, thus there is no sign change of MF wave-function

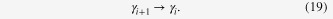
Therefore, we obtain the following rules for MFs evolution after exchanging positions of vortices hosting them

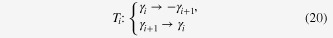
with *T*_*i*_ the braiding operation, consistent with our results of braiding MFs (13).

The unitary operator 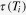 obeying 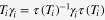 with *T*_*i*_ defined in (20) is given by [28]

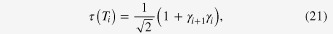
implying non-Abelian statistics. To demonstrate this, we consider four vortices hosting four MFs 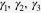 and 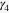, which combine into two complex fermions 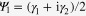 and 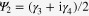. The unitary operators describing two ways of braiding MFs on basis 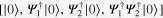 (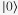 is the empty state) are [28]

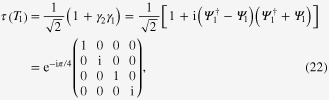


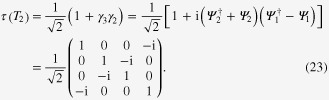
The non-Abelian statistics is then verified by the noncommutative relation of unitary operators 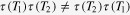 [3].

Direct manipulations of vortices might be done by using a STM tip, but suffer great difficulties. This is not only because that large effective masses of vortices make transportations of themselves hard and time-consuming, but also it is almost impossible to return vortices to exact positions in order to form a closed braiding loop, which would cause systematic errors in topological quantum computations. On the contrary, in our scheme of braiding MFs, vortices stay pinned at their original positions and thus no motion of vortices is necessary. What we do is tuning gate voltages at small regions connecting holes during braiding processes (see figures 3(a) and 4). It is guaranteed that MFs exchange their positions exactly after braiding.

### 4.2. Braiding MFs in a nanowire network

A toy model for realizing MFs in a *N*-site 1D spinless p-wave SC was proposed by Kitaev [8]

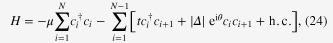
with *μ* the chemical potential, *t* nearest neighbor hopping rate, *Δ* superconducting gap function with phase *θ*. In the special case 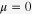 and 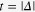, Hamiltonian (24) reduces to [8]

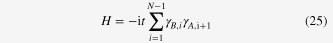
with

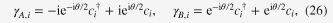
which are Majorana operators satisfying the self-adjoint condition 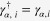, 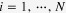 is the lattice site index of the 1D system. Observing that the two terms 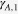 and 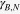 do not appear in the Hamiltonian (25), it can be concluded that the system can support two zero-energy quasi-particle states localized at two ends of the 1D SC (site 1 and *N*), while quasi-particles at other sites bind into complex fermions with finite energies. The two end states are just MFs, which appear in the topological phase for 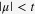 and disappear in the non-topological phase with 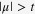 [8, 42].

**Figure 7.**
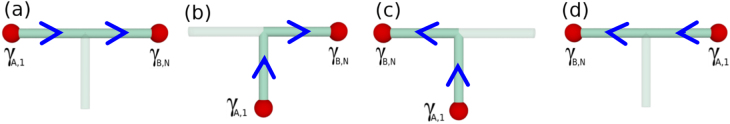
Schematic diagram for braiding MFs 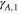 and 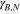 in a T-junction nanowire network. Green lines are nanowires in the topological phase and can support MFs (red balls). Light green lines are in the non-topological phase. Rightward/upward blue arrows indicate superconducting phase 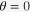, whereas leftward/downward arrows stand for 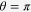.

In 2D spinless 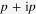 SCs, MFs bound to vortex cores catch nonzero Berry phases while moving around other vortices, which give rise to non-Abelian statistics by exchanging vortices [28]. At first glance, it seemed impossible to realize non-Abelian statistics by exchanging Majorana states in 1D systems since vortices are absent, and two MFs collide with each other and fuse into finite energy states while exchanging them. Alicea *et al* proposed to interchange MFs in a T-junction nanowire network (see figure 7) and proved that the exchanging of MFs follows non-Abelian statistics [41]. They realize Kitaevʼs toy model by putting a SM nanowire with strong spin–orbit coupling on top of an s-wave SC [20, 21, 27, 42]. For simplicity, we adopt the Kitaev model to demonstrate braiding processes in the following.

The authors consider two segments of 1D nanowires forming a T-junction [41], as shown in figure 7. Nanowire in the topological phase (green line) can be tuned into non-topological (light green line) region by adjusting local chemical potential *μ* using local gate voltage. To exchange positions of MFs in the T-junction (see figure 7), the authors first initialize the horizontal nanowire into topological state [41], hosting MFs 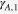 and 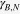 at the two ends (see figure 7(a)). The vertical nanowire is in the non-topological phase. By driving left part of horizontal nanowire into non-topological state and then vertical one into topological state, 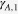 is transported to the bottom of vertical nanowire (see figure 7(b)). Next, 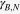 at the right end is moved to the left end of horizontal nanowire (see figure 7(c)). Finally, 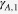 is moved to the right end of the horizontal nanowire (see figure 7(d)), which finishes interchanging of MFs. To track how 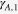 and 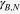 evolve after braiding, one can evaluate Berry phases acquired by the groundstate wave-function in principle. Here we adopt an alternative way to understand the statistics followed by interchanging MFs.

The topological energy gap of the 1D system remains nonzero while manipulating two MFs, which gives us the freedom to perform unitary transformations for Hamiltonian (24). For simplicity, we put Hamiltonian (24) to purely real while manipulating 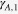 and 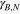, which is only possible when superconducting phase *θ* is either 0 or *π*. The phase of the pairing function depends on the direction of site index *i* assignment since 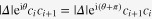. We denote 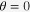 by rightward/upward arrows, then 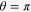 must be represented by leftward/downward arrows (see figure 7). After exchanging two MFs, we end up with reversed arrows along the topological nanowire 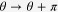, indicating the sign flipping of uniform superconducting pairing *Δ*. To restore Hamiltonian back to its original form, we multiply a phase factor 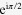 to fermionic creation operator 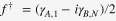 where *θ* is absorbed into

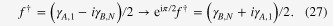
The above transformation gives

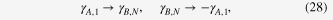
which generates the non-Abelian statistics same as braiding of vortices in 2D spinless 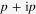 SCs as discussed above [28].

**Figure 8.**
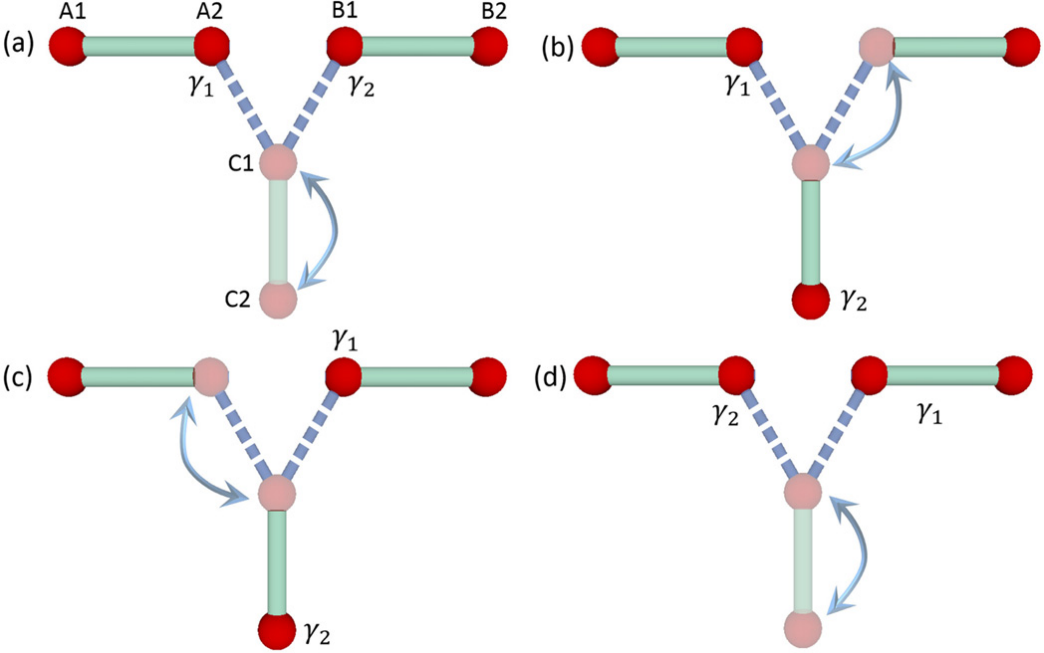
Schematic diagram for braiding MFs 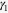 and 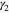 (red balls) at positions A2 and B1 respectively. Light blue arrows indicate finite couplings between MFs. Dashed blue lines are topological trivial segments.

There are other possible schemes for realizing non-Abelian statistics in 1D nanowire system [24, 26, 43, 44]. Sau *et al* [24] proposed to transport MFs by tuning couplings between MFs, instead of driving MFs all the way along the T-junction by tuning chemical potential largely [41]. The Hamiltonian describing couplings between two MFs is

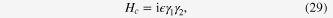
which can form a complex fermion 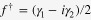 with finite energy. The number operator of fermion 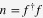 has double-degenerated groundstates 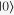 and 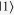 for 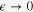. An energy gap *ϵ* is created between eigenstates 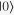 and 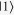 for finite *ϵ*. In order to exchange MFs, three nanowires are put close to each other, and MFs at positions C1 and C2 are allowed to couple together, forming a complex fermion with finite energy (see figure 8(a)). To transport MF 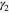, the authors adiabatically reduce the coupling between MFs at C1 and C2, and then increase that between B1 and C1. At zero coupling between C1 and C2, 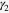 is moved to position C2 (see figure 8(b)). Next, 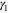 at A2 is transported to B1 (see figure 8(c)). Finally, 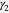 is moved to position A2 to complete the interchanging of MFs (see figure 8(d)). The trajectories followed by 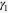 and 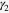 are

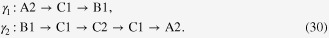
It is proven that interchanging of 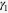 and 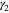 in this way follows non-Abelian statistics since either 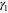 or 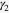 acquires a minus sign after exchanging positions [24].

In order to realize the above two braiding methods in nanowire networks [24, 41], some difficulties need to be overcome. In the proposal by Alicea *et al* [41], manipulations of MFs by using gate voltages become quite difficult in 1D system since one has to adjust a number of local gate voltages precisely in the whole network. Failing to exert correct gate voltage at a single lattice site may break down the whole braiding process. Besides, transportations of MFs through T-junctions depend on details of the junction, which may be difficult to control [24, 38]. As for the scheme by Sau *et al*, the coupling *ϵ* between MFs is oscillatory in space in topological SCs as well as upon changing chemical potentials [39], which is not easy to be controlled accurately in any macroscopic way, meaning that transportations of MFs can hardly be carried out in designed ways. In contrast, our proposal for manipulating MFs only requires tuning of local gate voltages at very small regions connecting two holes (see figure 3) and does not involve microscopic control on the couplings between MFs, which makes our scheme of braiding MFs robust.

## 5. Conclusion

We have shown that the MFs hosted by vortex cores in topological SCs can be liberated from pinned vortices, transported and braided over the prepared holes, taking advantages of the heterostructure of s-wave SC and spin–orbit coupled SM. By solving the TDBdG equation numerically, we monitor the time evolutions of MF wave-functions and demonstrate the non-Abelian statistics of adiabatical braidings of MFs. The present scheme only requires local applications of gate voltages, and minimizes possible disturbances to the MFs, which might be a challenging issue in other proposals based on end MFs in 1D SCs where gate voltages are necessary along the whole system. Instead of exchanging positions of vortices hosting MFs, our scheme of braiding operation is much easier and faster for experimental realizations of non-Abelian statistics. As compared with the edge MFs in 2D topological SCs, the core MFs are protected by a larger energy gap, which relaxes the limitation on operating temperatures. Therefore, the present scheme provides a more feasible way for manipulating MFs.
